# Effect of street trees on local air pollutant concentrations (NO_2_, BC, UFP, PM_2.5_) in Rotterdam, the Netherlands†

**DOI:** 10.1039/d4ea00157e

**Published:** 2025-02-06

**Authors:** Juliane L. Fry, Pascale Ooms, Maarten Krol, Jules Kerckhoffs, Roel Vermeulen, Joost Wesseling, Sef van den Elshout

**Affiliations:** a Meteorology and Air Quality (MAQ), Environmental Sciences Group, Wageningen University 6708PB Wageningen the Netherlands juliane.fry@wur.nl; b Institute for Marine and Atmospheric Research (IMAU), Utrecht University 3584 CC Utrecht the Netherlands; c Institute for Risk Assessment Sciences (IRAS), Division of Environmental Epidemiology, Utrecht University 3584 CM Utrecht the Netherlands; d National Institute for Public Health and the Environment (RIVM) 3720BA Bilthoven the Netherlands; e DCMR Environmental Protection Agency Rijnmond 3112NA Schiedam the Netherlands

## Abstract

Urban street trees can affect air pollutant concentrations by reducing ventilation rates in polluted street canyons (increasing concentrations), or by providing surface area for deposition (decreasing concentrations). This paper examines these effects in Rotterdam, the Netherlands, using mobile measurements of nitrogen dioxide (NO_2_), particulate matter (PM), black carbon (BC), and ultrafine particulate matter (UFP). The effect of trees is accounted for in regulatory dispersion models (https://www.cimlk.nl) by the application of an empirically determined tree factor, dependent on the existence and density of the tree canopy, to concentrations due to traffic emissions. Here, we examine the effect of street trees on different pollutants using street-level mobile measurements in a detailed case study (repeated measurements of several neighboring streets) and a larger statistical analysis of measurements across the urban core of Rotterdam. We find that in the summertime, when trees are fully leafed-out, the major short-lived traffic-related pollutants of NO_2_ and BC have higher concentrations in streets with higher traffic and greater tree cover, while PM_2.5_ has slightly lower concentrations in streets with higher tree factor. UFP shows a less clear, but decreasing trend with tree factor. In low-traffic streets and in wintertime (fewer leaves on trees) measurements confirm the importance of leaves to pollutant trapping by trees, by finding no enhancement of NO_2_ and BC with increasing tree cover, rather a slightly decreasing trend in pollutant concentrations with tree factor. Our observations are consistent with the dominant effect of (leafed-out) trees being to trap traffic-emitted pollutants at the surface, but that PM_2.5_ in street canyons is more often added by transport from outside the street, which can be attenuated by tree cover. Overall, these measurements emphasize that both traffic-emitted and regional sources are important factors that determine air quality in Rotterdam streets, making the effect of street trees different for different pollutants and different seasons.

## Abstract 2

Urban street trees can affect air pollutant concentrations by reducing ventilation rates in polluted street canyons (increasing concentrations), or by providing surface area for deposition (decreasing concentrations).

## Graphical abstract

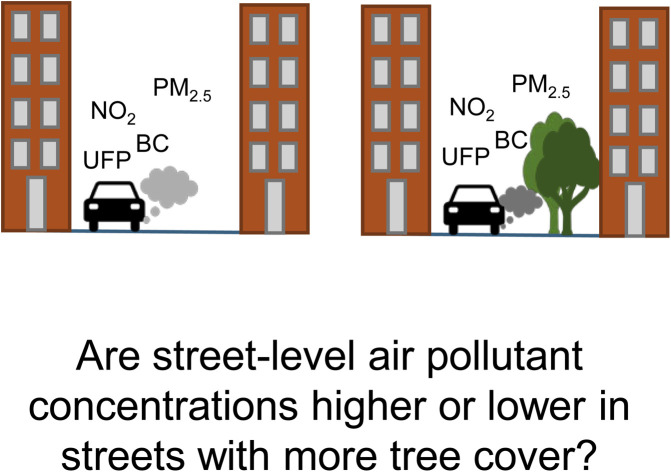

**Environmental significance**

Urban planners often include street trees in neighborhood designs to provide greenery and shade, with the understanding that this improves the well-being of city dwellers. In terms of air quality, however, the addition of trees can either improve air quality (by absorbing, filtering or excluding pollutants) or degrade it (by slowing wind speeds and thus reducing the ventilation that removes air pollution from street canyons). In this study, we examine the effect of street tree coverage on air pollutant concentration, using a new street-level mobile monitoring dataset collected across the urban core of Rotterdam, Netherlands. We compare summertime and wintertime observations to investigate the importance of the presence of leaves on the trees, and stratify the dataset by traffic level in the streets in order to determine the effects of trees with heavy and with light traffic. We find that leafed-out street trees enhance the street-level concentration of NO_2_ and black carbon, but have either no effect or even reduce the street-level concentration of particulate matter. The observational results are compared to the model used by the Dutch government to assess compliance with EU air quality directives. The results of our work can help inform future studies to determine the best placement of street trees to improve (or at least not deteriorate) air quality when planting for shade.

## Introduction

Around the world, populations are shifting towards urban areas, creating demand for new housing stock in cities. As new housing developments are built, urban planners make decisions about neighborhood design elements such as street width, building height, and the placement of street trees to create liveable communities. There are many dimensions of liveability, including thermal comfort, noise abatement, and aesthetics. A less visible dimension of liveability, also influenced by street design, is air quality.

**Fig. 1:**
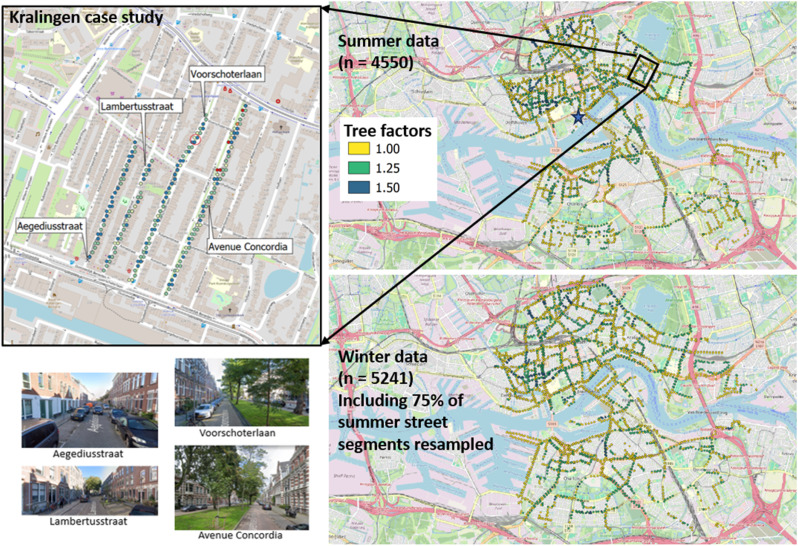
(Left) Map of 4 individual streets in the Kralingen neighborhood selected for the case study, with photos illustrating their street design and tree cover. Individual measurement points from one sampling day are shown to illustrate the sampling frequency and spatial density of samples during mobile measurements; one representative 30 m buffer is shown (red circle) to illustrate the size relative to this typical sampling density. (Right top) Full urban core summertime dataset used for the statistical analysis, showing the 4550 points at which measurements can be compared to modeled concentrations at the receptor sites. Points are colored by tree factor to show the distribution of tree density across the city (yellow = 1 [*n* = 2764], green = 1.25 [*n* = 1500], blue = 1.5 [*n* = 286]). (Right bottom) Full urban core wintertime dataset used for the statistical analysis, showing the 5241 points at which measurements can be compared to modeled concentrations at receptor sites. Points are again colored by tree factor (yellow = 1 [*n* = 3138], green = 1.25 [*n* = 1811], blue = 1.5 [*n* = 292]). In the upper right figure, the box indicates the location of the 4-street Kralingen case study, and the star indicates the location of the reference monitoring station at Rotterdam Schiedamse Vest. The wintertime sampling included 75% of the summer sampled streets.

Air quality is influenced by street design and trees because street canyons have their own airflow patterns. Trees provide leaf surface area that may absorb some pollutants, but can also slow wind speeds and thus reduce dispersion. This creates the so-called “green paradox,” wherein the well-intended introduction of urban greenery can either improve or degrade air pollution levels,^1–3^ sometimes even showing opposite trends within one city (*e.g.* street canyon *versus* open areas in Nanjing^4^). In complex urban areas, such as the city and port of Rotterdam, air pollutants have multiple sources, so that street-level pollution levels are affected by both local sources within the street canyon as well as transport into the street from other roadways and sources. In Rotterdam, sources include city roads, the freeway ring around the city, shipping traffic along the Maas River that bisects the urban core, and the port of Rotterdam to the west of the city (see Fig. 1). Especially for longer-lived pollutants such as PM_2.5_, we therefore expect influx from sources outside the street canyon to contribute a substantial fraction of street-level pollutant concentrations.

Different air pollutants can have varying interactions with (leaf) surfaces and thus may be differently affected by street trees.^5,6^ They also have different atmospheric lifetimes: from a few hours for ultrafine particulate matter to and NO_2_,^7,8^ to a few days to weeks for fine particulate matter. These pollutants also have varying health effects,^9^ with ultrafine particulate matter recently emerging as a pollutant exposure with significant association cardiovascular and respiratory mortality.^10,11^ We focus here on the traffic-related air pollutants nitrogen dioxide (NO_2_), black carbon (BC), ultrafine particulate matter (UFP), and particulate matter smaller than 2.5 microns (PM_2.5_), in street canyons of various designs. Mobile sampling using fast-response research-grade instruments in a moving vehicle enables the efficient collection of data across space, allowing the sampling of diverse street environments across an urban area.^12,13^

Air pollution dispersion models can account for street design variations by applying factors related to street type and tree cover to receptor site concentrations. The Dutch government calculates annually averaged concentrations of air pollutants at receptor sites along major roadways, as driven by emissions, surface topography, and meteorology. One parameter included in this calculation is the tree cover factor at each receptor point. These calculations are conducted to assess compliance with the European Air Quality Directive (task: environmental law, “Wet milieubeheer” or “Omgevingswet”), and are run at the level of the both national and local authorities.^14^ The latest EU Air Quality Directive identified priority pollutants to include NO_2_, PM_1_, PM_2.5_, PM_10_, but not BC or UFP.

Our aim in this study is to test two hypotheses about the effect of street trees on (various) pollutant concentrations: (1) the dominant effect of increasing tree cover is to reduce ventilation in street canyons, thus increasing street-level pollutant concentrations, and (2) the dominant effect of increasing tree cover is to increase available surface area for pollutant filtration, thus decreasing street-level pollutant concentrations. The current regulatory models assume hypothesis (1) is dominant for all pollutants, and apply a multiplicative tree factor in the CIMLK model, which increases the traffic contribution to all pollutant concentrations proportionally to tree cover. In this study, we compare the expected effect of tree cover on the modeled street-level concentrations of the criteria pollutants NO_2_, PM_2.5_, and elemental carbon (EC), to the measured street-level concentrations of four combustion related pollutants (NO_2_, PM, BC, and UFP), to investigate differences in how each pollutant is affected by trees.

## Methods

### Ruisdael Rotterdam and RI-URBANs 2022 measurements in Rotterdam

From 22. August – 9. September, 2022, the Ruisdael Observatory (https://www.ruisdael-observatory.nl/) organized a field campaign in Rotterdam (https://www.ruisdael-observatory.nl/measurement-campaign-maps-ghg-emissions-and-air-pollution-in-rotterdam/), with researchers studying urban atmosphere interactions *via* an intensive 3 weeks of measuring trace gases and aerosol around the city and port of Rotterdam. In November and December 2022, as part of the RI-URBANs project, street-level air pollution was sampled in Rotterdam with the same vehicle over 22 days of sampling. The spatial coverage of these summer and winter datasets are shown in Fig. 1; the sampling dates and wind conditions are listed in the ESI (Table A0).†

### Mobile monitoring of air pollutants

During these measurement campaigns, we deployed an instrumented car from Utrecht University^12^ equipped with a fast (50 liters per minute) inlet manifold and multiple pollutant monitors to sample >80 hours each season, driving the streets of Rotterdam and around the Rotterdam harbor. The car was equipped with fast-response instrumentation to measure NO_2_ (Aerodyne Research, Inc. CAPS monitor), BC (Magee Scientific AE33 aethalometer), UFP number count (TSI EPC 3783), and PM_2.5_, (TSI DustTrak), all at 1 second time resolution. A Global Positioning System (GPS, G-Star IV, GlobalSat, Taiwan) recorded the car's location at high time resolution, and the data were aligned by date and time. Measurements were conducted between 6 am and 6 pm local time on all days of the week, covering various parts of the city on different days.

### Centraal instrument monitoring luchtkwaliteit (CIMLK) air quality model

The Dutch RIVM uses a model (described in detail at https://www.rivm-syso.github.io/CIMLK/) to calculate highly-resolved spatial maps of annual average pollutant concentrations at receptor sites along major streets. Based on global background pollution maps (Grootschalige Concentratiekaarten Nederland (GCN)), the model uses known point and mobile emissions, typical meteorology, and accounts for relevant features such as trees and surface roughness, to determine the annual average concentration per substance at each receptor site.

The primary receptor characteristic analyzed in this study is tree factor (“BOOM_FACT”), describing the street segment tree cover. The tree factor variable options are 1 (no trees or only occasional trees), 1.25 (one or more rows of trees, less than 15 m apart and with openings between the crowns), and 1.5 (trees with crowns touching and covering at least one-third of the street width). This factor is applied only to the concentration contribution due to traffic. These tree factors are described in more detail and with figures in the documentation for the Dutch Standard Calculation Method 1 (SRM-1, Section 3.3 for BOMENFACTOR, https://www.rivm.nl/bibliotheek/rapporten/2014-0127.pdf). The tree factor is applied as a positive multiplicative factor, which increases pollutant concentrations in streets with high tree cover.

For each receptor site, the CIMLK reports annual average pollutant concentration for a subset of the measured species (NO_2_, EC, PM_2.5_). We download receptor characteristics and modeled concentrations from the CIMLK website, using the latest available Monitoring sonde of 2021, selecting the calculated annual average for 2020 (CIMLK Monitoring sonde 2021, Monitoringsjaar 2020: https://www.cimlk.nl/documentatie/downloads/nsl-downloads/MR2021/Wegverkeer/j2020/). Because the model reports all concentrations in μg m^−3^, we converted from observed ppb NO_2_ to μg m^−3^ NO_2_ at 293 K and 101.3 kPa atmospheric pressure, using a conversion factor of 1 ppb = 1.9 μg m^−3^. The CIMLK elemental carbon (“EC”) variable is related to black carbon (BC) by the empirical relationship EC = 0.4 × BC; modeled EC values are converted to BC using this relationship to enable comparison with measurements.

### Case study and big-data strategies to comparing streets

Two distinct strategies were used to investigate differences between pollutant concentration in streets with differing tree cover. First, a case study was conducted to compare adjacent streets of differing tree cover in the Kralingen neighborhood. The four selected streets (street 1 = Avenue Concordia, has trees separating 2 lanes, 2 = Voorschoterlaan, has trees separating 2 lanes, 3 = Lambertusstraat, few trees with one-way traffic in a single lane, 4 = Aegidiusstraat, no trees with one-way traffic in a single lane) were sampled 6 times during the Ruisdael Rotterdam campaign in August 2022, each time sampling all the streets in sequence over the course of about 15 minutes, to minimize differences in background conditions. These streets were chosen for their similar traffic but contrasting street design, and the fact that they are parallel, ensuring similar winds in the streets if consecutively sampled.

Second, the full 3 weeks (15 days) mobile monitoring August/September summer dataset and 22 days November/December winter dataset, trimmed to the residential urban core of Rotterdam, was used for two seasonal large dataset statistical analyses. Circular buffers of 30 m diameter were constructed around each CIMLK model receptor site and all mobile measurements within those buffers were aggregated to that point. For every point for which there exist between 3 and 200 measurements, the mean concentration of each measured pollutant was calculated. The minimum of 3 was set to avoid using sampling points where we passed so quickly that few measurements were made; the maximum of 200 was set to exclude points where the car may have been stationary, for example behind another vehicle, and thus sampling the effects of congestion rather than street design. The buffer size of 30 m was chosen so that data collected in the middle of the street will be assigned to the nearest receptor points (see red circle in upper left panel of Fig. 1).

To focus on the effect of trees specifically on traffic-related pollutants, we use available traffic data to filter this dataset to the 50% highest and 50% lowest traffic receptor points. Traffic data is also available in the CIMLK database, aggregated per street segment and reported as annual average vehicle counts per day. We used QGIS to join to each receptor point the geographically nearest traffic data. Based on the distribution of traffic at the selected receptor sites, the high/low traffic filtering means dividing the dataset for passenger vehicle counts (“INT_LV_mea” in CIMLK) above and below 3574 vehicles per day.

These two comparison strategies are complementary; in the case study, the close spatial and temporal proximity of the sets of measurements lessens the likelihood of variability in other confounding variables during the sampling (*e.g.* meteorological conditions, time of day), but it only has a small number of samples. In the second, big-data approach, the aggregation of all measured data within a spatial buffer of each receptor point creates a large dataset (>4000 samples) over multiple days of sampling, increasing background meteorological variability but lessening the likelihood of the results being skewed by *e.g.* individual vehicle plumes.

Fig. 1 shows maps of the case study streets in the Kralingen neighborhood of Rotterdam (left), with photos illustrating the distinction between street designs and tree cover, and on the righthand side, a full map of all receptor points used in the summertime and wintertime big dataset statistical analysis.

## Results and discussion

### Case study: Kralingen

**Fig. 2:**
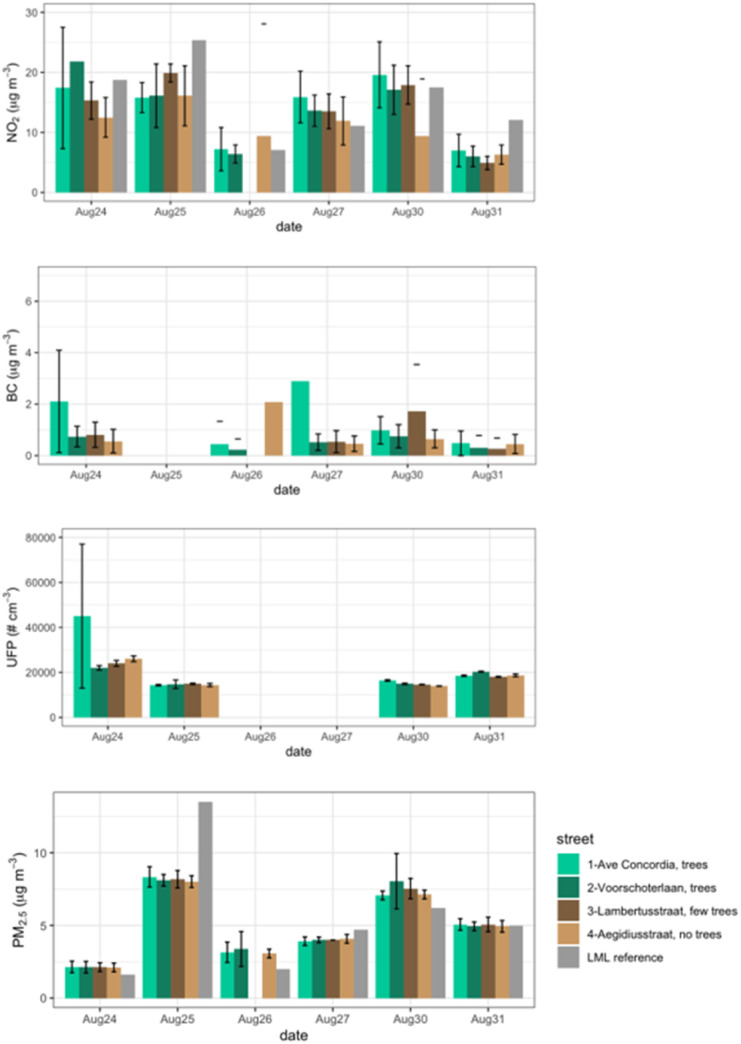
Means (whiskers: ±1 standard deviation) of pollutant concentrations observed in each of the four streets sampled in close temporal proximity on six dates in August 2022. Table 1 lists the number of measurements per street section; there were at least 100 measurements in each street segment. Across the 6 days of sampling, concentrations of UFP, BC, and NO_2_ are typically higher in the streets with trees (Avenue Concordia and Voorschoterlaan) than those without (Lambertusstraat and Aegidiusstraat), with the difference most pronounced and consistent for BC and NO_2_. For PM_2.5_ and NO_2_, reference data is available from the Schiedamse Vest urban background monitoring site 2.5 km to the southwest of the Kralingen neighborhood measured, and the measured values for the closest hour to the sampling period is shown in the figure as the grey bars.

Fig. 2 presents a clustered bar chart of the mean and standard deviation of concentrations of each pollutant in each street segment, alongside the concentrations measured at the closest reference monitoring station (Landelijke Meetnet Luchtkwaliteit (LML), Rotterdam Schiedamse Vest urban background monitoring site 2.5 km to the southwest of the measured streets). A summary of all six sampling days is shown in Table 1. We present the data as means, medians, and standard deviations per street segment so that the reader can visually assess the (lack of) differences between street segments.

**Table 1:** Case study of repeated measurements in 4 Kralingen, Rotterdam streets. nd = no data available due to instrument error. Green shaded lines are the higher tree cover streets. The bottom line reports available national air quality measurements network (LML Rotterdam Schiedamse Vest site) reported value is the hourly average for the hour closest to the Kralingen sampling on this date (car timestamp is UTC)

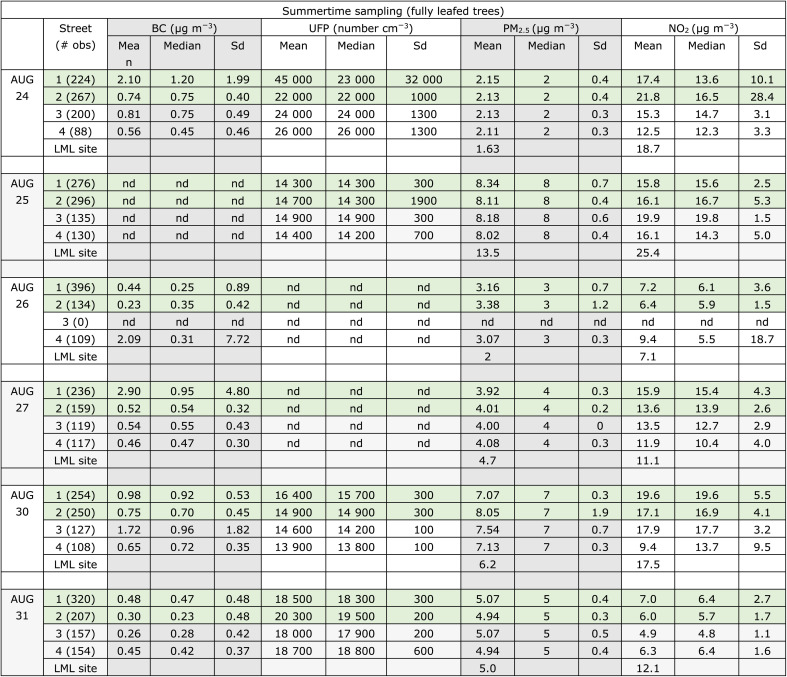

We observe (Fig. 2 and Table 1) that most days, pollutant concentrations were somewhat higher in the streets with trees (Avenue Concordia and Voorschoterlaan, green shading in figure and table) than those without (Lambertusstraat and Aegidiusstraat, brown shading in figure). The greatest street-to-street differences, showing higher concentrations in tree-covered streets than bare streets, were seen for NO_2_ and BC. Less contrast was observed for UFP and PM_2.5_, for which concentrations were almost never significantly different across streets. Fig. 2 shows the mean values for each street ±1 standard deviation error bars, with overlapping error bars in many cases illustrating that these are not strong trends. We also note that data are missing on some sampling days due to instrument errors.

The comparison of mobile measured concentrations to LML reference monitoring data across sampling days can give a sense of whether day to day trends are regionally representative. Indeed, we see that day-to-day variability is largely consistent between the Kralingen streets and the LML reference station, with the exception of Aug. 25, which had substantially higher measurements at the urban background site than in the Kralingen neighborhood. In these streets, due to relatively light traffic, the NO_2_ and PM_2.5_ mean concentrations are regionally driven, with only small differences potentially attributable to street-to-street differences.

The general pattern of (slightly) higher concentrations of pollutants in the tree-lined streets relative to the bare streets is consistent with the leafy trees slowing wind speeds and therefore reducing ventilation in those streets. However, differences in recent traffic could also influence observed concentration differences.

The Kralingen streets are differentiated in Fig. 2 with their qualitative tree cover (trees, few trees, no trees), but it is also important to note that there are other differences: Lambertusstraat and Aegidiusstraat have a single lane of traffic and are 11 to 15 m wide, while Concordia Avenue and Voorschoterlaan have 2 lanes with trees between and are 25 to 26 m wide. Based on personal observations, the traffic during sampling was not dramatically different across the four streets, but the large variability and small differences in mean here illustrate the limitations of this case study. To better quantify this effect, and to complement this detailed Kralingen case study, we turn to a statistical analysis of the full Rotterdam urban core dataset.

### Statistical analysis of full mobile monitoring datasets (summer and winter)

We analyzed a full dataset of 4550 (summer)/5241 (winter) locations within the urban core of Rotterdam, as described in the Methods section above. We report the results divided into higher-traffic and lower-traffic halves in order to test our hypotheses about potential tree effects specifically on traffic-emitted pollutants.

### Observed and modeled concentrations binned by tree factor, split by traffic

**Fig. 3:**
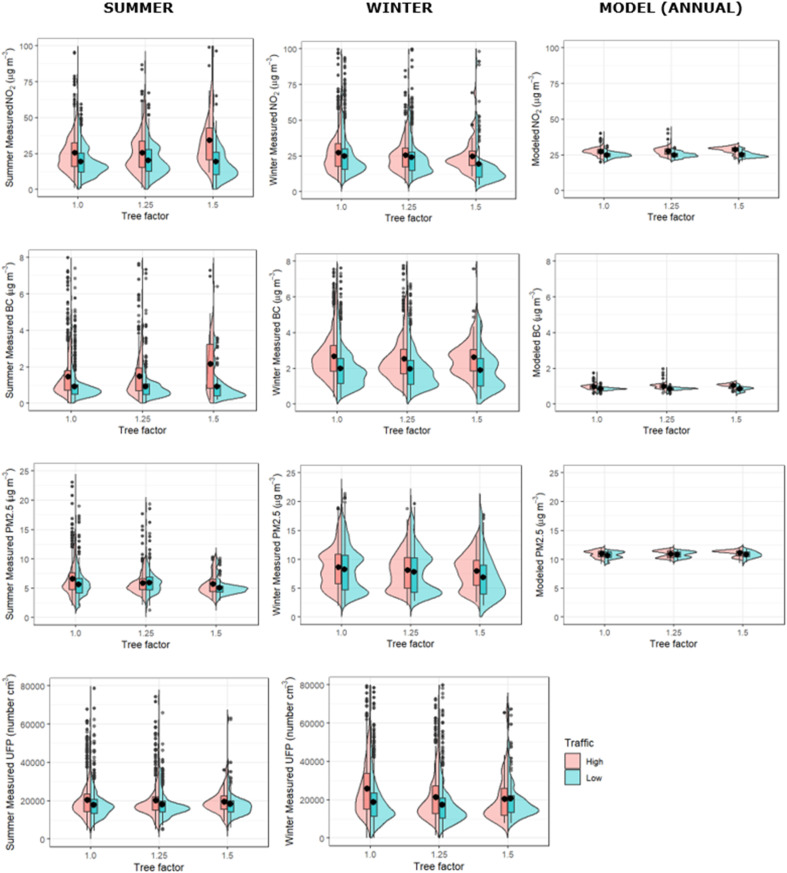
Measured and modeled NO_2_, BC, and PM_2.5_, binned by tree factor, split into the highest and lowest 50% traffic counts at the receptor sites. Note that the summer observations were all made in August and September, 2022, and the winter observations in November and December 2022, while the modeled concentrations are the predicted annual average for 2020. UFP is not modeled in CIMLK so only the measurements are shown.

The maps on the righthand side of Fig. 1 show the distribution of tree factors over the streets analyzed in this full dataset. All three tree factor classes are well represented, although with fewer data points in the densest tree cover category of 1.5. The pollutant concentration comparisons across varying tree factor, for each season, divided into high and low traffic halves, are shown as split violin plots in Fig. 3. For each receptor site, there is a CIMLK model annual average pollutant concentration for NO_2_, EC, and PM_2.5_. These modeled pollutant concentrations are, binned and displayed in the same way as a function of tree factor, next to the corresponding observations in Fig. 3.

Table 2 presents means, medians, and standard deviations for measured and modeled pollutant concentrations, for the high-traffic half of the summer dataset, binned by tree factor. Table 3 presents these same statistics but for the low-traffic half of the summer dataset. Tables 4 and 5 repeat these statistics for the wintertime dataset.

Summertime pollutant data (mean, median, and standard deviation) aggregated by tree factor, filtered to the 50% highest traffic receptor sites. Modeled concentrations are shown at right for comparison; note these modeled values are annual averages. Variability (expressed as standard deviation) is large in the measured data due to real environmental variability during the campaign, nevertheless the upward trend in mean and median concentrations with increasing tree cover is clearly present in this high traffic data for NO_2_ and BC, while PM_2.5_ mean and median concentrations slightly decrease with increasing tree cover, and UFP has no clear trend

**Table:** 

Tree factor	Measured NO_2_ (μg m^−3^)			Modeled NO_2_ (μg m^−3^)		
	Mean	Median	Std dev	Mean	Median	Std dev
1	25.5	23.5	13.5	27.3	27.4	2.8
1.25	25.8	23.4	14.1	27.4	27.8	2.6
1.5	41.2	34.4	29.1	28.5	29.4	2.4

**Table:** 

Tree factor	Measured BC (μg m^−3^)			Modeled EC, converted to BC (μg m^−3^)		
	Mean	Median	Std dev	Mean	Median	Std dev
1	1.91	1.11	3.16	0.98	0.98	0.13
1.25	2.07	1.16	4.16	0.99	1.00	0.12
1.5	2.37	2.09	1.89	1.03	1.05	0.13

**Table:** 

Tree factor	Measured PM_2.5_ (μg m^−3^)			Modeled PM_2.5_ (μg m^−3^)		
	Mean	Median	Std dev	Mean	Median	Std dev
1	6.96	5.86	4.40	11.1	11.2	0.58
1.25	5.92	5.43	2.43	11.1	11.3	0.59
1.5	5.70	5.27	1.85	11.2	11.3	0.51

**Table:** 

Tree factor	Measured UFP (# cm^−3^)					
	Mean	Median	Std dev			
1	21 600	18 200	15 400			
1.25	21 700	17 700	17 200			
1.5	19 400	18 000	5600			

Summertime pollutant data aggregated by tree factor, in this case filtered to the 50% lowest traffic receptor sites. Modeled concentrations are shown at right for comparison; note these modeled values are annual averages. Variability (expressed as standard deviation) is large in the measured data due to real environmental variability during the campaign, nevertheless we see that, in contrast to Table 2, for these streets with lower traffic source, we do not observe an increase in NO_2_ or BC concentrations with increasing tree cover. Both PM_2.5_ and UFP concentrations decrease (slightly) with increasing tree cover

**Table:** 

Tree factor	Measured NO_2_ (μg m^−3^)			Modeled NO_2_ (μg m^−3^)		
	Mean	Median	Std dev	Mean	Median	Std dev
1	19.4	16.7	10.7	24.6	24.8	2.1
1.25	20.5	17.9	11.9	25.1	24.8	2.1
1.5	19.3	14.5	14.4	25.3	25.1	2.1

**Table:** 

Tree factor	Measured BC (μg m^−3^)			Modeled EC, converted to BC (μg m^−3^)		
	Mean	Median	Std dev	Mean	Median	Std dev
1	0.95	0.73	0.91	0.84	0.85	0.08
1.25	0.96	0.71	1.03	0.86	0.87	0.09
1.5	0.89	0.60	0.91	0.84	0.87	0.12

**Table:** 

Tree factor	Measured PM_2.5_ (μg m^−3^)			Modeled PM_2.5_ (μg m^−3^)		
	Mean	Median	Std dev	Mean	Median	Std dev
1	5.74	5.42	2.73	10.8	11.0	0.6
1.25	5.96	5.84	2.02	11.0	11.1	0.5
1.5	5.08	4.92	1.30	10.9	10.9	0.5

**Table:** 

Tree factor	Measured UFP (# cm^−3^)					
	Mean	Median	Std dev			
1	20 300	16 900	24 100			
1.25	19 700	16 500	9800			
1.5	18 300	16 800	7700			

Wintertime pollutant data (mean, median, and standard deviation) aggregated by tree factor, filtered to the 50% highest traffic receptor sites. Modeled concentrations are shown at right for comparison; note these modeled values are annual averages and thus identical to Table 2. Variability (expressed as standard deviation) is large in the measured data due to real environmental variability during the campaign, nevertheless it is clear that the summertime upward trend in mean and median concentrations with increasing tree cover is not present in the wintertime data, rather, all mean and median pollutant concentrations (NO_2_, BC, PM_2.5_ and UFP) slightly decrease with increasing tree cover

**Table:** 

Tree factor	Measured NO_2_ (μg m^−3^)			Modeled NO_2_ (μg m^−3^)		
	Mean	Median	Std dev	Mean	Median	Std dev
1	27.3	25.0	14.1	27.3	27.4	2.8
1.25	25.7	23.6	12.3	27.4	27.8	2.6
1.5	24.6	22.3	10.6	28.5	29.4	2.4

**Table:** 

Tree factor	Measured BC (μg m^−3^)			Modeled EC, converted to BC (μg m^−3^)		
	Mean	Median	Std dev	Mean	Median	Std dev
1	2.79	2.52	1.51	0.98	0.98	0.13
1.25	2.57	2.25	1.34	0.99	1.00	0.12
1.5	2.62	2.65	1.06	1.03	1.05	0.13

**Table:** 

Tree factor	Measured PM_2.5_ (μg m^−3^)			Modeled PM_2.5_ (μg m^−3^)		
	Mean	Median	Std dev	Mean	Median	Std dev
1	8.61	8.30	3.47	11.1	11.2	0.58
1.25	8.16	8.00	3.55	11.1	11.3	0.59
1.5	7.96	7.89	2.93	11.2	11.3	0.51

**Table:** 

Tree factor	Measured UFP (# cm^−3^)					
	Mean	Median	Std dev			
1	26 300	22 700	15 000			
1.25	22 000	17 600	14 900			
1.5	21 300	15 600	14 300			

Wintertime pollutant data aggregated by tree factor, in this case filtered to the 50% lowest traffic receptor sites. Modeled concentrations are shown at right for comparison; note these modeled values are annual averages and thus identical to Table 3. Concentrations are all slightly lower than the high traffic, but the trends are similar to the high traffic wintertime data, with all pollutants except UFP generally decreasing slightly with increasing tree cover

**Table:** 

Tree factor	Measured NO_2_ (μg m^−3^)			Modeled NO_2_ (μg m^−3^)		
	Mean	Median	Std dev	Mean	Median	Std dev
1	25.5	20.5	15.5	24.6	24.8	2.1
1.25	24.2	20.7	14.1	25.1	24.8	2.1
1.5	22.0	14.1	22.7	25.3	25.1	2.1

**Table:** 

Tree factor	Measured BC (μg m^−3^)			Modeled EC, converted to BC (μg m^−3^)		
	Mean	Median	Std dev	Mean	Median	Std dev
1	2.04	1.78	1.28	0.84	0.85	0.08
1.25	2.02	1.74	1.31	0.86	0.87	0.09
1.5	1.89	1.82	1.08	0.84	0.87	0.12

**Table:** 

Tree factor	Measured PM_2.5_ (μg m^−3^)			Modeled PM_2.5_ (μg m^−3^)		
	Mean	Median	Std dev	Mean	Median	Std dev
1	8.28	8.00	3.89	10.8	11.0	0.6
1.25	7.87	7.50	3.79	11.0	11.1	0.5
1.5	6.87	5.95	3.67	10.9	10.9	0.5

**Table:** 

Tree factor	Measured UFP (# cm^−3^)					
	Mean	Median	Std dev			
1	19 400	15 500	14 000			
1.25	18 800	14 400	15 300			
1.5	21 500	16 500	14 200			

The first observation is that in the high-traffic summertime subset (Fig. 3 and Table 2), the measured NO_2_ trend from no trees (tree factor = 1) to some trees (tree factor = 1.25) to dense trees (tree factor = 1.5) is a substantial increase in both mean and median concentration, while in the low-traffic subset (Fig. 3 and Table 3) there is no such trend, even with the lowest concentration measured at highest tree factor. Black carbon shows a similar pattern, with generally increasing concentrations with increasing tree cover on high-traffic streets, and no trend/lowest concentration at highest tree factor on low-traffic streets. These observations are consistent with leafy trees hindering NO_2_ and BC dispersion in high-traffic streets, while in lower-traffic streets, where traffic emissions are less important than transport of background pollution from elsewhere in the urban region into the street canyon, the (slightly) lower observed NO_2_ and BC concentrations at the highest tree cover suggest that trees can filter or prevent pollutants from elsewhere to enter these streets, overcoming any reduction in dispersion they cause.

In contrast, for PM_2.5_ (and, albeit less significantly, UFP), the observed summertime mean and median concentrations decrease slightly with increasing tree factor, in both the low-traffic and high-traffic data subsets. In wintertime, concentrations are overall higher and the decreasing trend with increasing tree factor is a bit more visible. This is consistent with the major sources of these pollutants most often being outside the street canyon, and thus the dominant effect of increasing tree cover is always filtering or excluding this pollution. This reduction of regional PM pollution entering the street overcomes the decrease in ventilation caused by trees, even in the high-traffic streets. The fact that this trend is also observed in wintertime either means it is not simply an effect of the trees (perhaps instead reflecting different emissions in the sampled streets), or could mean that the exclusion of particulate matter from street canyons by trees does not strongly depend on whether or not those trees are leafed-out.

We note that the CIMLK-modeled annual-average pollutant concentrations always increase with increasing tree factor, due to the model assumption that the main impact of trees is to reduce ventilation. Note that in Wesseling (2016)^15^ it was observed that the effect of the tree-factor in the model may be too large. There was not enough data on different combinations of street types and amounts of trees to draw definite conclusions. The modeled trend is larger for NO_2_ and BC than for PM_2.5_, because of the larger traffic contribution to NO_2_ and BC. However, such an increasing trend of pollutant concentration with increasing tree cover was observed only in the high-traffic summertime dataset, and only for the pollutants NO_2_ and BC, and was substantially more pronounced than the modeled trend.

A possible physical explanation for the difference in trends between NO_2_ and BC *versus* PM_2.5_ is that the main sources of NO_2_ and BC to a street canyon are the vehicles on-road underneath the tree canopy, causing the main effect of trees to be a reduction of ventilation and thus increase in concentration, while PM_2.5_ arrives primarily by transport from outside the street, and in this case the more important (but smaller magnitude) effect of trees is to filter or exclude this external incoming air, thus either not affecting or reducing PM concentrations as tree cover increases. Rotterdam has diverse sources of PM_2.5_ from industrial and shipping activity in the Port of Rotterdam, in addition to all the typical urban sources.

The fact that UFP has almost no trend across tree factor in summertime makes it difficult to interpret. The lack of increased pollutant concentration with increasing tree cover highlights the dynamical behavior of UFP, which we expect to have large local point emissions (for example diesel scooters) as well as in-transport of new particle formation from upwind precursors. More study is needed to understand the impact of trees on street-level concentrations of UFP.

We note that wintertime concentrations are generally higher, which is consistent with mixing of pollutants into a shallower boundary layer driven by colder surface temperatures. We also note larger variability (standard deviations) in BC and PM_2.5_ concentrations in wintertime relative to summertime. We suspect this is due to the winter sampling (22 days in November and December 2022) sampling a greater variety of meteorological background conditions and thus boundary layer heights. Table A0 in the ESI† reports the average wind speed and direction for each sampling day; while the wind direction spans all directions in both season, the wind speed has substantial larger variability (expressed as the standard deviation) in winter than in summer. We note that because each monitoring drive day sampled many streets of each tree factor, day-to-day differences in these background conditions will not have introduced any systematic bias in the tree factor trends.

The fact that in wintertime a slight decrease is observed in most pollutant concentrations with increasing tree factor could be interpreted in two ways: (1) the presence of trees even without leaves acts to filter or exclude transport of pollutants into street canyons, or (2) there is a baseline difference in pollutant emissions across these sets of streets, against which the summertime trends can be compared to observe the effects of leaves on trees. If the first assumption is true, we might expect a stronger decrease in PM_2.5_ and UFP with increasing tree cover in summer than winter, when leaves would be expected to enhance the filtering/exclusion effects of trees on PM. This is not clearly the case, but it is difficult to assess, because both the means and variability in pollutant concentrations are larger in winter. If the second assumption is true, the summertime ventilation reduction enhancement of street-level NO_2_ and BC under increasing tree cover is even stronger than the observed trend, since it overcomes this baseline trend in the opposite direction. Regardless, the aggregate data tells us that under leafy trees, street-level NO_2_ and BC are enhanced only in the summertime, while PM_2.5_ and UFP concentrations are either unaffected or slightly decreased by the presence of (leafy) trees.

### Comparing the magnitudes of observed and modeled concentrations

Lastly, we compared the absolute magnitudes of the overall measured and modeled pollutant concentrations. Here we note two key caveats: (1) the measurements were made in either August and September (summer) or November and December (winter), while the modeled concentrations are reported as annual averages, and (2) the measurements were made in the center of the traffic lane, above the front windshield of the sampling car, while the modeled concentrations are calculated for the sidewalk, at which distance the concentrations will be slightly lower. Studies have shown these differences are usually between 10 and 30%, depending on the pollutant.^12,16^ Since we focus primarily on comparisons across streets, this difference is less important. The seasonal nature of the measurements *vs.* the annually averaged model outputs explains the much lower variability in all modeled concentrations.

NO_2_ concentrations are typically expected to be higher in winter, when boundary layer height is lower, and the shorter daylight shifts the NO/NO_2_ equilibrium towards NO_2_. However, we observe modeled (annual average) NO_2_ concentrations to be slightly higher than all measured concentrations, except summertime observations in the highest-tree streets, which are substantially larger than modeled. The observed high to low traffic differences are larger than modelled across both seasons. However, in general, the modeled concentrations more closely match those measured for NO_2_ than the other pollutants. The results of the CIMLK model have been extensively compared to yearly average PM_2.5_, PM_10_, NO_*x*_ and NO_2_ concentrations.^15,17^

We observe even larger contrast between high- and low-traffic streets in measured BC concentrations, especially in summer (and increasing with tree cover), but also in winter. Observed concentrations are up to double (triple) the modeled concentrations in high traffic streets in summer (winter), and 10–20% higher in the low-traffic streets in summertime and more than double in low-traffic streets in winter. These higher than modeled BC concentrations, especially pronounced in high-traffic streets, suggests that the BC emissions of the current vehicle fleet may be larger than estimated. We remind the reader that we have used the approximate empirical relationship of EC = 0.4 × BC to convert modeled concentrations to compare to BC; this conversion factor may have a seasonal variation due to contributions of wood burning in winter, but if anything is likely to be larger in winter,^18^ which would only increase the difference between measurements and modeled concentrations.

Observed PM_2.5_ is lower than modeled in both summertime and wintertime, by approximately a factor of two in summer and by ∼30% in winter, when higher concentrations are likely driven by lower boundary layer height. We note that the PM_2.5_ mobile measurements were not made by a reference-grade instrument and should be interpreted cautiously. We refer to the monthly average measured PM_2.5_ concentrations at the Rotterdam Schiedamse Vest monitoring site in central Rotterdam (the nearest site to the measurements used here) in 2021 and 2022. Summer (May through September) concentrations are indeed lower than winter (November through March), but the seasonal differences appear less pronounced than the difference between observed summertime and wintertime concentrations seen here. At Rotterdam Schiedamse Vest in 2022, the mean PM_2.5_ summer and winter concentrations for 2021/2022 were 9/8 and 11/13 μg m^−3^, respectively, while our summer and winter observations (in high/low traffic streets) were 6.5/6 and 8.5/8 μg m^−3^, respectively. These measurements from the monitoring site show more seasonal contrast as well as higher typical values than measured during our campaign. Because the individual day comparisons of PM_2.5_ measurements to this monitoring station show good agreement (Fig. 2 and A1†), we conclude that the days of our measurements campaign (August 22–September 9, 2022) had lower than normal summertime PM concentrations.

### Measurement trend uncertainties, sampling caveats, and recommendations for future work

It is important to note that we are interpreting the average behavior across a large dataset in streets with many additional factors beyond tree cover, like instantaneous traffic and meteorology, varying. Thus, we are careful to interpret trends only as being “consistent with” certain tree effects, and not as providing definitive evidence of those effects. The standard deviations reported alongside the mean and median pollutant concentration values in Tables 1–5 are intended to enable the reader to interpret the (non)significance of all discussed trends.

The comparison to LML data shown in Fig. 2 confirms that much day-to-day background variability in concentration is captured by both stationary monitoring sites and mobile measurements. A more systematic comparison of all mobile measured data points near regulatory monitoring stations compared to the coincident monitoring station measurements (shown in ESI Fig. A1†) further confirms that the mobile measurements match stationary monitoring stations when they are nearby and coincident.

With regard to the trends shown in Fig. 3, we note that the underlying assumption in a “big data” approach of representativity of the dataset is not assured. The randomized driving of Rotterdam streets in both summertime and wintertime achieved coverage of a large number of receptor data points (see Fig. 1), of which 75% were sampled in both seasons. However, there may be correlations between traffic intensity and tree cover that could affect the observed trends. We checked the large datasets for biases due to the timing of the sampling, and for bias in the types of streets with different tree factors. The details of these checks are shown in the ESI Tables A1–A3.† One notable trend is that tree factor = 1 by definition often correlates with the street type classification that has no buildings near either side of the roadway, which would act to further facilitate ventilation in these cases. In Rotterdam on the whole, the mean traffic load is not identical across different tree factors, but the average tree factor is the same for busy and calm traffic roads. Our overall conclusion from these checks is that neither sampling timing in different streets nor potential relationships between street types and tree cover would change the qualitative trends, but due to potential confounding factors such as variable instantaneous traffic and meteorological conditions during sampling, we do not interpret the tree factor trends quantitatively.

Based on these identified concerns about trend uncertainties and sampling representativeness, we make recommendations for future mobile monitoring studies. In order to enable quantitatively interpreting tree cover trends, it would be best to have a large enough dataset to isolate streets of a certain type and traffic level before comparing pollutant levels and different tree factors. To reduce the impact of instantaneous variations in traffic, it would also be valuable to sample these representative streets multiple times. Thus, for a targeted study of the effect of tree cover on pollutant concentrations, a hybrid of the Kralingen case study and big data approaches may be the best strategy: select a large subset of representative streets with a single street type and several consistent strata of traffic intensity, but varying tree cover, and then sample these streets repeatedly over multiple sampling days with varying background meteorological conditions.

## Conclusions

The current general consensus, that the dominant effect of street trees is to slow wind speeds, thus reducing ventilation and increasing surface pollutant concentrations, was confirmed in our study for NO_2_ and BC. Substantially higher concentrations in streets with greater tree cover were found in the summertime, consistent with an important role of the tree canopy in trapping (some) pollutants at the street surface. This hypothesis is supported by the absence of elevated concentrations in high tree cover streets in the wintertime data.

In contrast, PM_2.5_ shows a different pattern, with slightly higher concentrations in treeless streets and lower concentrations in the streets with greater tree canopy cover. This is consistent with either the baseline emissions being higher in the treeless streets, or with the major source of PM to urban streets coming from outside the street canyon itself, and the possibility that some of this incoming PM can be filtered and removed by the tree canopy. The fact that wintertime observations find a flat to slightly decreasing trend in all pollutant concentrations with increasing tree cover (more pronounced for PM_2.5_ and UFP) is consistent either with this being the baseline trend in emissions, which the summertime enhancement in high-tree streets overcomes only for NO_2_ and BC, or with all pollutants having a small sink to tree surface area, regardless of the presence of leaves. UFP shows a more mixed pattern, presumably due to the combination of local traffic sources and transport of new particle formation plumes *e.g.* from the port of Rotterdam.

These results show that the CIMLK model approach of calculating yearly average concentrations for receptor sites misses some interactions that could have substantial annual variability, in particular, the effect of tree cover. Our results suggest that whether street tree cover increases or decreases pollutant concentrations depends on the season, the pollutant identity, and on the traffic level, with the first effect (reducing ventilation = increase) dominating for high-traffic streets and for the pollutants NO_2_ and BC, while the second effect (pollutant filtering = decrease) may dominate for PM and UFP under all traffic conditions. It would be of interest to identify streets with different types of tree cover, to investigate potential air pollutant concentration differences caused by specific tree types or the structure of the tree canopy.

Regardless of the effects of trees on pollutant concentrations, it is essential to remember that trees provide highly beneficial shade, water retention, and aesthetic amenities to all streetscapes, which may outweigh any detrimental effects on air quality. However, the trapping of pollutants below tree canopies could be mitigated for example by spacing trees further apart to reduce the tunnel effect. Finally, we wish to emphasize that the most effective way to improve street-level air pollution is not to modify the trees, but rather to reduce or remove the combustion vehicle sources of those pollutants. Because the pollutant trapping effects are most pronounced in summer, policies reducing traffic during summertime would be especially beneficial.

## Data availability

Data used in this study include publicly available tree factor, traffic data, and receptor modeled pollutant concentrations from the Dutch Ministry of Environment and Public Health's Centraal Instrument Monitoring Luchtkwaliteit at https://www.cimlk.nl/. Data collected by the mobile vehicle for this project are available *via* discussion with the coauthors, and will be made publicly available at the end of the projects at https://www.riurbans.eu/results/ and https://www.ruisdael-observatory.nl/data/.

## Author contributions

J. F.: conceptualization, formal analysis, investigation, methodology, supervision, project administration; writing – original draft; P. O.: investigation, writing – reviewing & editing; M. K.: investigation, writing – reviewing & editing; J. K.: funding acquisition, data curation, resources, writing – reviewing & editing; R. V.: funding acquisition, resources, writing – reviewing & editing; J. W.: resources, writing – reviewing & editing; S. V. D. E.: resources, writing – reviewing & editing.

## Conflicts of interest

There are no conflicts to declare.

## Supplementary Material

EA-005-D4EA00157E-s001
