# Supplementary material for: Effect of street trees on local air pollutant concentrations (NO2, BC, UFP, PM2.5) in Rotterdam, the Netherlands
Source: Environ Sci Atmos. 2025 Feb 6;5(3):394–404. doi: 10.1039/d4ea00157e (PMC11844741; doi:10.1039/d4ea00157e)
Supplement: EA-005-D4EA00157E-s001 [file EA-005-D4EA00157E-s001.pdf]

This journal is © The Royal Society of Chemistry 2025

Supplemental Information

Day-to-day variation in meteorological background conditions (wind direction and windspeed) appear more variable in the winter than in summer, helping explain the larger observed variability in measured pollutant concentrations in winter.

Table A0. Average wind speed and direction on all sampling days. Data was obtained from https://www.daggegevens.knmi.nl/klimatologie/uurgegevens using weather station 344, Rotterdam, and averaged from 6 am to 6 pm on each sampling day. Overall, we see similar mean wind speeds in both seasons, but greater variability (standard deviation) in winter. Dominant wind directions are mixed in both seasons.

**Sampling date (summer)**	**Wind speed (m s^{-1})**	**Wind direction**
22. Aug 2022	2.9	ESE
23. Aug	2.9	WSW
24. Aug	2.5	SSW
25. Aug	3.1	ESE
26. Aug	4.5	NNW
27. Aug	3.2	N
28. Aug	3.8	NNE
29. Aug	2.2	NNE
30. Aug	5.9	ENE
31. Aug	6.5	NE
1. Sep	4.4	E
2. Sep	4.6	E
4. Sep	2.8	S
5. Sep	3.6	SE
6. Sep	3	SSW
7. Sep	4.5	S
**Summer mean +/- stdev**	**3.8 +/- 1.2**	
**Sampling date (winter)**		
16. Nov 2022	5.2	SSE
20. Nov	3.2	ESE
23. Nov	6.5	S
25. Nov	4.7	SW
26. Nov	4.4	S
30. Nov	1.2	N
2. Dec	5.8	NE
5. Dec	3.5	NE
6. Dec	1.6	WSW
7. Dec	2.1	W
8. Dec	3.7	W
9. Dec	1.4	S

10. Dec	3.6	S
12. Dec	2.2	SSE
13. Dec	2.5	E
14. Dec	1.5	NNE
15. Dec	2.2	SSE
16. Dec	1.5	N
19. Dec	8	S
20. Dec	5.9	S
21. Dec	5	SSW
22. Dec	3.8	SW
**Winter mean +/- stdev**	**3.6 +/- 1.9**	

Comparisons of mobile measurements of $PM_{2.5}$ and NO_2 made within 500 m of a stationary monitoring site over the course of the Ruisdael summertime campaign confirm the overall match between the instruments measuring in the mobile vehicle and the regulatory (LML) reference stations (Figure A1). Note the greater scatter for the shorter-lived pollutant NO_2. We also note a greater tendency for NO_2 in-street values to exceed those measured at the monitoring station, giving a slope larger than 1.

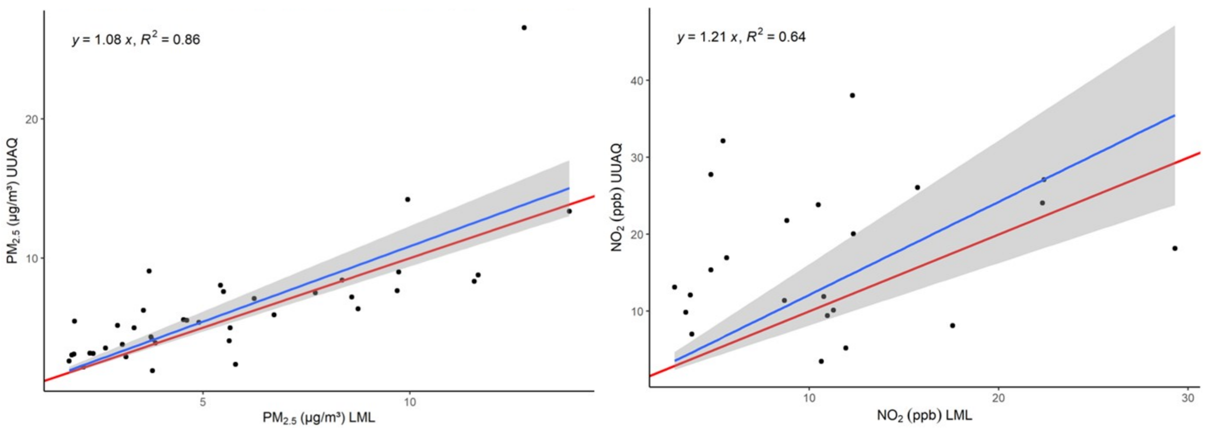

Figure A1. Comparison of (a) $PM_{2.5}$ and (b) NO_2 measurements made within 500 m of LML monitoring stations, compared the simultaneous measurement values at that station.

In order to check for potential bias in sample timing, we repeat the summertime trend analysis in the big dataset for a subset of sample timing that is characterized by stable concentrations of NO_2. We identify this time period by inspecting the hourly NO_2 concentrations values at the urban background monitoring site, Rotterdam Schiedamsevest, during the summertime measurement campaign dates in 2022 (see Figure A2). We observe relatively stable concentrations between the hours of 11-17:00 local time, and thus take this subset of hours from the dataset (this reduces the dataset size approximately by half).

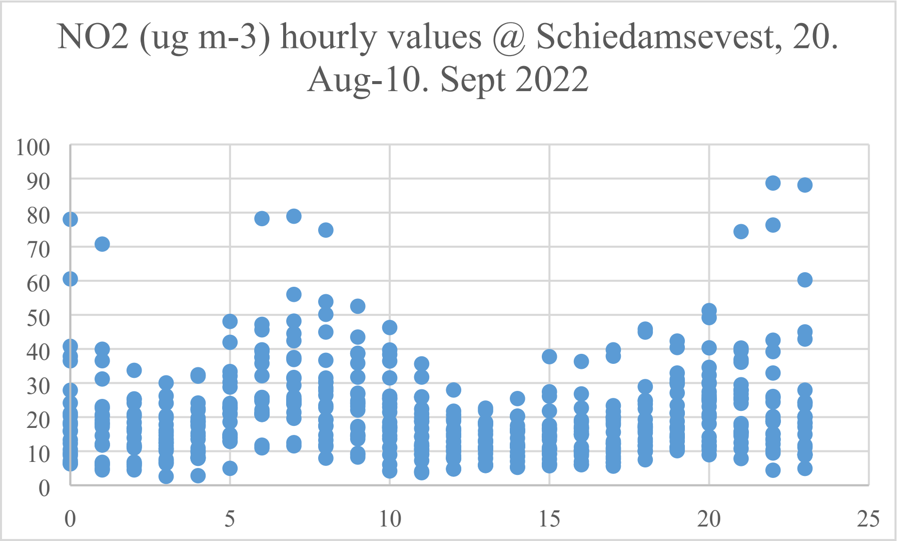

Figure A2. Hourly NO_2 concentration observed at Rotterdam Schiedamsevest monitoring station during the Ruisdael Rotterdam 2022 campaign. Data shows more or less stable concentrations from 11:00-17:00 daily, albeit with substantial day to day variability.

Now, we re-run the summertime analysis on this subset of the dataset, with results shown in Figure A3. Because we observe in this subset similar trends to the overall analysis, we conclude that there is no temporal bias in the sampling that affects the conclusions from the large dataset. The one exception is that UFP here appears to increase slightly with increasing tree cover under high-traffic conditions, rather than decrease slightly. However, we have already deemed UFP a mixed case without a clear trend, so this could be due to the UFP sources in the sampled streets being a relatively larger contribution during this period than the regional transport of UFP into those streets, while in the full dataset, more periods with dominant regional transport are sampled. In any case, this does not affect any conclusions of this paper.

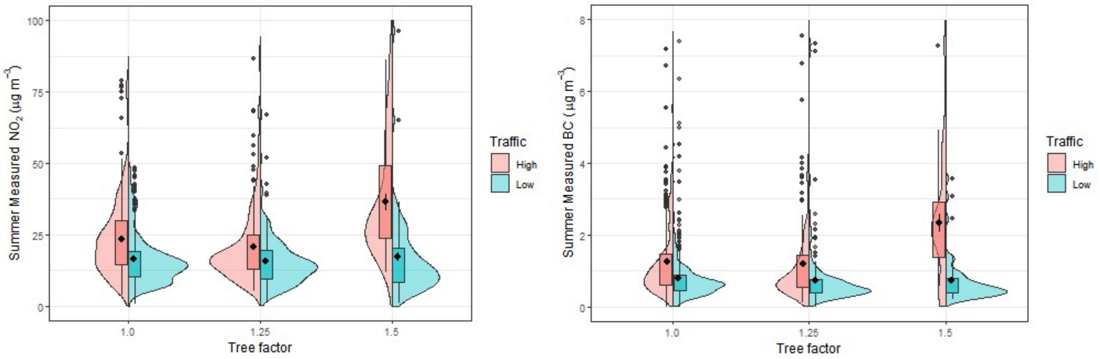

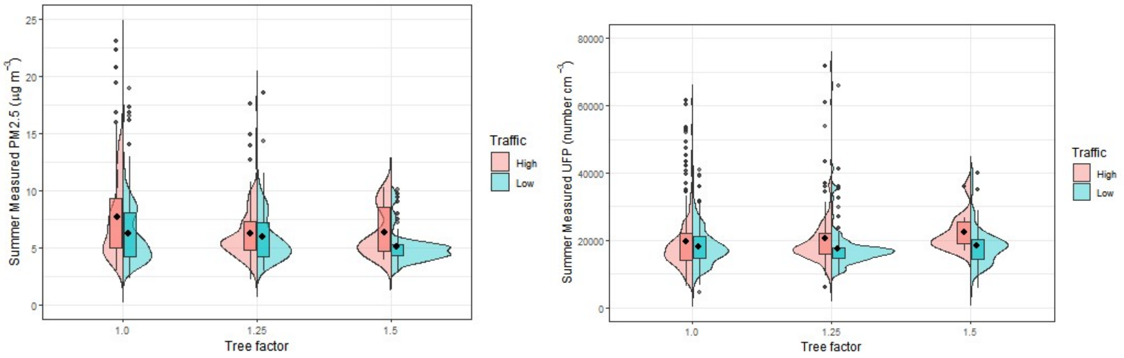

Figure A3. Summertime tree factor analysis (same as lefthand panels of Figure 4), repeated for the 11-17:00 time subset of the data. The trends are similar, resulting in the conclusion that the dataset it not significantly time-biased.

In order to check for potential biases in street types sampled under different traffic or tree factor conditions, we check the street type ("WEGTYPE") distribution for each traffic condition and tree factor, to assess the similarity. The CIMLK definition of the 4 street types are shown in Figure A4, and the

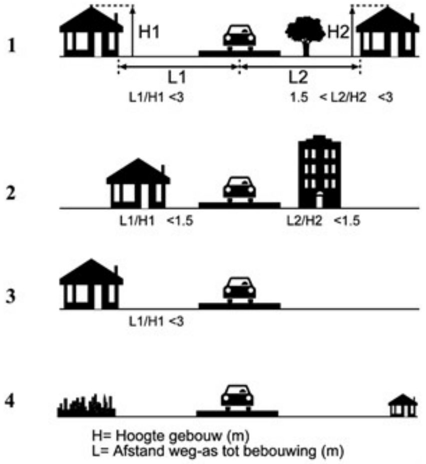

resulting distributions are shown below in Table A1.

Figure A4. Definitions of WEGTYPE (= street type) variables in CIMLK. Source: https://www.infomil.nl/onderwerpen/lucht-water/luchtkwaliteit/slag/monitoren-nsl/handleiding-monitoring-nsl/monitoringstool/bijlagen/rekenregels/

Table A1. Distributions of WEGTYPE for each season + traffic condition, and for each tree factor. The street type distributions are largely similar across season & traffic condition. We do note that the receptor points for treefactor = 1 are more heavily skewed towards street type 4, which by definition has no nearby buildings on either side, which would further increase ventilation.

WEG TYPE	Summer high traffic	Summer low traffic	Winter high traffic	Winter low traffic	treefactor = 1	treefactor = 1.25	treefactor = 1.5
1	428	360	539	420	365	359	94
2	366	656	429	585	638	297	88
3	544	570	651	555	693	353	70
4	899	715	1223	808	1064	521	34

Table A2. Examining traffic load (vehicles per day) by tree factor for all receptor sites in the Rotterdam city center included in the CIMLK database. No linear relationship between tree factor and traffic is observed, but the highest tree factor streets have on average less traffic. This means the summertime increases in NO_2 and BC at 1.5 may be even higher if traffic were taken into account.

Tree factor	Mean traffic load (excluding heavy vehicles)	Stdev of traffic load	Number of receptor sites
1	4448	4475	6567
1.25	4994	4628	3445
1.5	3639	3586	496
all	4589	4500	10508

Table A3. Now splitting the above dataset in half by mean traffic load, and checking the average tree factor for busy (traffic > 4589 vehicles per day) versus calm (traffic < 4589 vehicles per day) streets. We find virtually the same average tree factor across traffic, suggesting that similar tree factors are represented across high and low traffic streets for the entire Rotterdam urban core (similar to what we observe for our subset of sampled receptor sites in Table A1).

Traffic	Mean traffic load (excluding heavy vehicles)	Mean tree factor	Stdev of tree factor	Number of receptor sites
busy (> 4589 veh day^{-1})	9035	1.11	0.14	4176
calm	1656	1.10	0.15	6332

(< 4589 veh day^{-1})				
all	4589	1.11	0.15	10508
